# A prospective, longitudinal study of growth, nutrition and sedentary behaviour in young children with cerebral palsy

**DOI:** 10.1186/1471-2458-10-179

**Published:** 2010-04-06

**Authors:** Kristie L Bell, Roslyn N Boyd, Sean M Tweedy, Kelly A Weir, Richard D Stevenson, Peter SW Davies

**Affiliations:** 1 Queensland Cerebral Palsy and Rehabilitation Research Centre, Discipline of Paediatrics and Child Health, School of Medicine, The University of Queensland, Brisbane, Australia; 2 Children's Nutrition Research Centre, Discipline of Paediatrics and Child Health, School of Medicine, The University of Queensland, Brisbane, Australia; 3 Queensland Children's Medical Research Institute, Brisbane, Australia; 4 The University of Queensland, School of Human Movement Studies, Brisbane, Australia; 5 Department of Speech Pathology, Royal Children's Hospital, Brisbane, Australia; 6 Kluge Children's Rehabilitation Center & Research Institute, University of Virginia (UVA) Children's Hospital, UVA School of Medicine, Charlottesville, Virginia, USA

## Abstract

**Background:**

Cerebral palsy is the most common cause of physical disability in childhood, occurring in one in 500 children. It is caused by a static brain lesion in the neonatal period leading to a range of activity limitations. Oral motor and swallowing dysfunction, poor nutritional status and poor growth are reported frequently in young children with cerebral palsy and may impact detrimentally on physical and cognitive development, health care utilisation, participation and quality of life in later childhood. The impact of modifiable factors (dietary intake and physical activity) on growth, nutritional status, and body composition (taking into account motor severity) in this population is poorly understood. This study aims to investigate the relationship between a range of factors - linear growth, body composition, oral motor and feeding dysfunction, dietary intake, and time spent sedentary (adjusting for motor severity) - and health outcomes, health care utilisation, participation and quality of life in young children with cerebral palsy (from corrected age of 18 months to 5 years).

**Design/Methods:**

This prospective, longitudinal, population-based study aims to recruit a total of 240 young children with cerebral palsy born in Queensland, Australia between 1^st ^September 2006 and 31^st ^December 2009 (80 from each birth year). Data collection will occur at three time points for each child: 17 - 25 months corrected age, 36 ± 1 months and 60 ± 1 months. Outcomes to be assessed include linear growth, body weight, body composition, dietary intake, oral motor function and feeding ability, time spent sedentary, participation, medical resource use and quality of life.

**Discussion:**

This protocol describes a study that will provide the first longitudinal description of the relationship between functional attainment and modifiable lifestyle factors (dietary intake and habitual time spent sedentary) and their impact on the growth, body composition and nutritional status of young children with cerebral palsy across all levels of functional ability.

## Background

Cerebral palsy (CP) is the most common cause of physical disability in childhood occurring in 1 in 500 children [1]. It is a group of permanent disorders of movement and posture, causing activity limitation, that are attributed to non-progressive disturbances that occurred in the developing foetal or infant brain [2]. Damage to the structure of the brain is static and permanent; however, the consequent symptoms are variable and may change over time [2]. In addition to disordered movement or posture, children may have a range of associated disabilities, including intellectual disability, hearing and visual deficits, nutrition, feeding and swallowing problems, respiratory infections and epilepsy [1]. Cerebral palsy has substantial life long effects on daily function and quality of life (QOL) for children and their families with an estimated economic cost of over AUD $115,000 per person per annum [3].

### Growth and nutritional status of children with CP

Poor growth and nutritional status are commonly reported in children with CP [4,5]. Conversely, there is evidence to suggest that certain children with CP are at risk of obesity, particularly those with marked spasticity and who are relatively inactive [6]. Poor growth is frequently considered a 'normal', untreatable side-effect of CP, however, the impact of poor growth on health, participation and QOL is an area that requires further investigation [7]. Most studies have concentrated on severely impaired children and are frequently flawed by a lack of valid and repeatable methods for assessing linear growth and body composition in this population [8].

The largest study to date into the growth parameters of children and adolescents with CP was based on retrospective data relating to height and weight obtained from the patient records of 24,920 children and adolescents aged 2 - 20 years. The 10^th^, 50^th ^and 90^th ^percentile curves for body weight, height and body mass index (BMI) were developed from over 141,900 measurements of weight and height [8]. This study confirmed that children with moderate to severe motor impairment are growth impaired. Trends for lower weight and height for age were apparent for the lower functioning groups and deviated further from those of the general population with increasing functional impairment. The largest differences in weight and height were seen in those with the most severe motor impairment. Interestingly, in the lowest functioning groups (groups 4 and 5), the presence of a feeding tube was associated with greater weight and height (group 5), in comparison to children in group 4 who did not have a feeding tube. The major strengths of this study were the large sample and the development of growth curves stratified by gross motor skills and mode of feeding. The most significant limitations of the study were that the methods utilised to measure height were of unknown validity and reliability in this population, non validated tools were used to determine functional severity and the sample was largely cross sectional with only a portion having repeated measures.

Importantly, the growth curves presented in this study are purely descriptive of growth within the study population and have not been related to health outcomes [8]. Any representative sample of children with CP will include a large number of undernourished subjects, as such, these population specific growth charts are not a prescription for desirable growth in this group. This study raises two key questions related to growth and nutrition for children with CP: what is desirable growth and, what is the relationship between growth, nutritional status and health related outcomes and QOL in this population?

### Causes of poor growth in CP

It has been hypothesised that poor growth in children with CP may be related to nutritional factors, physical factors or factors related to the brain lesion itself. Nutritional factors include inadequate dietary intake, secondary to impaired oral motor and swallowing competence and poor nutritional status and may impact directly on growth [4,9,10]. Physical factors result in decreased mechanical stress on bones due to immobility or lack of weight bearing [11]. Bone growth studies have suggested that immobilisation decreases bone formation and longitudinal bone growth and increases bone resorption, which suppresses certain growth-stimulating hormones [11]. Factors related to the brain lesion itself may impact on growth either directly (via a negative neurotrophic effect on linear growth) or indirectly (via the endocrine system) [4,10]. Growth differences between impaired and unimpaired limbs in children with hemiplegia, support the hypothesis that non-nutritional factors play a significant role in reducing growth in children with CP [12].

Cross-sectional studies have identified links between feeding ability and measures of growth and nutritional status [13,14]. Longitudinal investigations have found that early nutritional supplementation by gastrostomy results in improved linear growth in children with severe CP if commenced early in life [10,15,16]. Swallowing difficulties have been reported in up to 99% of children with CP classified as Gross Motor Function Classification System (GMFCS) IV or V, the majority of which exhibit moderate to severe (76%) or profound (15%) dysphagia [17]. The prevalence of dysphagia in children with more mild motor impairment (GMFCS scores I-III) is unknown, as is the point at which oral motor dysfunction begins impacting on dietary intake and growth. Specific issues related to oral motor and swallowing problems in CP include poor saliva control and drooling [18]; difficulty sucking, chewing and swallowing [19-21]; and oropharyngeal aspiration [22-24], all of which impact on lifestyle. Poor saliva control has been associated with health and lifestyle impacts such as poor hygiene, reduced social acceptability of anterior drooling, reduced social interaction and self-esteem, increased daily cares [25-27] and aspiration of posterior drooling with associated pulmonary complications [28]. The impact of oral motor impairment on feeding and swallowing has been associated with reduced dietary intake leading to suboptimal nutritional status and requirement for tube feeding [13,14]. Other health issues related to oral motor and swallowing problems include pulmonary complications and pneumonia associated with oropharygneal aspiration requiring multiple hospitalisations [28-30], and lifestyle impacts on the child and family such as extended length of mealtimes [31].

In a sample of 171 children with CP, Stevenson and colleagues [4] found that children with severe gross motor impairment had significantly lower height *Z-*scores than less impaired children and that mid arm circumference and tricep skinfold thickness highly correlated with both height and weight *Z-*scores. This study suggests that growth is related to body composition and severity of CP. Stallings and colleagues [9] found that disease severity variables (oral motor function, ambulatory status, and gastrostomy feeding) and non-disease variables (age, pubertal status, gender, and mid parental height) explained approximately 70-75% of the variability in length of 142 children with quadriplegic CP. After controlling for these, body composition (upper arm muscle area and percent body fat) explained 10-15% of the remaining variation. The magnitude of the impact of body composition on linear growth was similar to that of disease severity. Importantly, body composition had a stronger effect on the growth of younger children compared to older children. Both of these studies were cross sectional and therefore the strength of evidence is low.

The cross-sectional multi-centred study North American Growth in CP Project (NAGCPP) showed a significant relationship between functional severity and nutritional status in a group of 235 moderately to severely impaired children (GMFCS III-V) [32,33]. These children, aged 2-18 years, had lower fat-stores, shorter stature, and decreased muscle mass compared to typically developing children. In addition, these studies demonstrated an association between overall growth status and increased health care use and impaired participation [7,32]. The NAGCPP did not include an entire population based sample, few children were less than 3 years and only children with moderate to severe motor impairment (GMFCS III-V [34]) were included. In addition, lifestyle factors (dietary intake and time spent sedentary) were not assessed.

### Physical activity and time spent sedentary in children with CP

Habitual physical activity is an established determinant of health and, in Australia, the cost of illness directly attributable to insufficient activity is AUD$377m per annum across the entire population [35]. In children, physical activity is required for healthy growth and development, including building strong bones and muscles, improving balance, and acquiring and developing motor skills [36]. The best available evidence indicates that people with mobility impairment are among the least physically active groups in society [37,38], and consequently children with CP may be at risk of sub-optimal growth and development secondary to physical inactivity. Unfortunately studies investigating the link between time spent sedentary and growth and development in young children with CP- particularly those who are unable to walk - have not been conducted. Studies which accurately document patterns of sedentary behaviour in this population and relate the data to health outcomes are urgently needed. Such studies require the development and evaluation of methods for assessing activity and inactivity in children who move in a range of different ways including crawling, cruising, rolling and bottom shuffling. Results will permit ascertainment of the importance of inactivity prevention and physical activity promotion strategies in the management of children with CP, as well as the identification of high need groups within the CP population.

### Difficulties with the assessment of growth and nutritional status in CP

The neuromuscular complications associated with CP make accurate anthropometric and body composition measurements difficult and sometimes impossible in this population. Our group, and others, have overcome this issue by using segmental limb measures which provide reliable, valid and clinically useful alternatives to measuring height in children with CP [39-41]. For evaluation of body composition, the use of deuterium-oxide is considered a "gold-standard-technique" due to its reliability, accuracy and the limited assumptions required with its use compared to other more commonly used and widely available measures such as skinfold thicknesses; however, its limited availability, cost and time required for analysis result in a technique that is generally prohibitive for routine clinical use. When used in combination with published hydration constants [42,43], deuterium-oxide can be used to determine fat free mass and hence fat mass in children, using the two component model of body composition. It is a safe, non-radioactive, naturally occurring, isotope that has been used to measure total body water in a wide range of groups including pregnant women, infants and the elderly [44,45].

Current investigations into the growth, oral motor and feeding difficulties and nutritional status of children with CP have focused on cross sectional data at one time point or diverse samples across a broad age range. They concentrate on only the most severely impaired children without use of validated measures of height, body composition, gross motor function and health status. Measures of feeding ability and oral motor dysfunction have been most commonly derived from parent questionnaires rather than the use of validated clinical tools. There have been no longitudinal investigations into the impact of lifestyle factors (dietary intake and time spent sedentary) on growth, body composition, nutritional status and their impact on health outcomes in children with CP. The paucity of such information reduces capacity to develop and implement effective management strategies for this population.

## Aims and hypotheses

**Figure 1:**
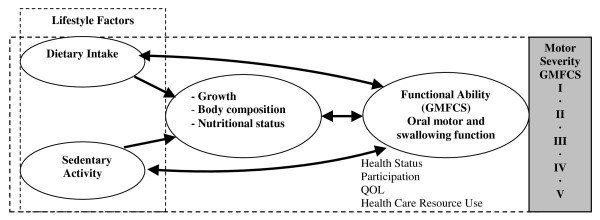
**Conceptual model illustrating the hypothesized interactions of the principle factors to be investigated in the prospective, longitudinal study of growth, nutrition and sedentary activity in young children with cerebral palsy**.

This study will investigate the influence of growth, body composition, dietary intake, oral motor and swallowing function and time spent sedentary (adjusted for motor severity) on health outcomes, participation and QOL in a prospective population based study of young children with CP (from corrected age (ca) of 18 months to 5 years). The hypothesized interaction between these factors is represented graphically in the conceptual model (see figure 1).

This broad aim will be addressed by the following four hypotheses (H):

### H1

Growth status, nutritional status and growth velocity, from 18 months of age will be related to the level of gross motor functional attainment (GMFCS) at 5 years of age.

### H2

Body composition (fat free mass and fat mass) will be related to the level of gross motor functional attainment at 5 years of age.

### H3a

For a given GMFCS level, dietary intake, oral motor/swallowing function and time spent sedentary at 3 and 5 years of age will be significantly related to growth velocity and body composition.

### H3b

The relationship between dietary intake, oral motor/swallowing function & time spent sedentary at 18 months will predict growth status, nutritional status and body composition at 5 years of age.

### H4

Controlling for functional severity, children with slower growth, suboptimal body composition (fat free mass and fat mass), lower levels of oral motor/swallowing function and greater time spent sedentary will have:

(i) higher health care utilisation and direct medical costs at 3 and 5 years.

(ii) lower levels of participation in school, leisure and community at 3 and 5 years.

(iii) poorer QOL at 5 years.

### Study significance

This study will be the first longitudinal, population based study to enable more accurate prediction of the early natural history of nutritional and growth problems in young children with CP linked to dietary intake, time spent sedentary, health outcomes and resource utilization. Specifically this project will:

• Determine the nature and timing of nutritional, feeding and growth abnormalities.

• Enable better prediction of the likelihood and impact of sub-optimal dietary intake from an earlier age.

• Enable planning of nutritional treatment options at optimal times.

• Develop and validate methods for measuring sedentary behaviour in young children with CP, including those who do not walk as their primary means of locomotion.

• Highlight the relative contribution of poor dietary intake, oral motor and feeding difficulties and sedentary behaviour on growth and body composition taking into account severity of disability.

• Quantify the impact of dietary intake and time spent sedentary on medical resource use to inform service provision planning.

• Define the relationship between habitual time spent sedentary and functional abilities to predict eventual functional attainment.

• Define the relationship between oromotor/swallow dysfunction and gross motor attainment.

• Quantify the impact of poor nutrition and high amounts of time spent sedentary on participation in society and QOL.

• Allow interpretation of data derived from clinical methods for the assessment of body size and composition (eg body mass index (BMI) and skin-fold thickness).

## Methods/Design

This prospective, population based longitudinal study aims to recruit a total of 240 young children with CP born in Queensland, Australia, between 1^st ^September 2006 and 31^st ^December 2009. It is being conducted in conjunction with another study: Queensland CP Child Study of Motor Function and Brain Development (NHMRC 465128). Ethics approvals have been received from the University of Queensland Medical Research Ethics Committee (2008002260), the Children's Health Services District Ethics Committee (HREC08/QRCH/112/AM01), the CP League of Queensland (CPLQ 2008/2010 1029), Gold Coast Health Service District Human Research Ethics Committee (HREC/09/QGC/88), and the Townsville Health Service District Human Research Ethics Committee (HREC/09/QTHS/96). Further ethics approvals are being sought from additional paediatric and regional centres throughout Queensland.

### Selection criteria

#### Inclusion criteria

All Queensland born children diagnosed with CP, born between 1^st ^September 2006 and 31st December, 2009. We define CP as a group of permanent disorders of movement and posture that are attributed to non-progressive disturbances that occurred in the developing foetal or infant brain [2]. The characteristic signs are spasticity, movement disorders, muscle weakness, ataxia and rigidity [46].

#### Exclusion criteria

Children with a progressive or neurodegenerative lesion will be excluded from the study.

### Recruitment

Recruitment for this study commenced in April 2009 and state-wide recruitment has been established in collaboration with the Queensland CP Register, the Queensland CP League, the Queensland Children's Health Services District, the Queensland CP Health Service, and other regional hospitals and health service districts throughout Queensland. Community awareness has been generated through paediatricians, general practitioners, allied health professionals, child health nurses, and neonatal follow-up clinics. These groups have been encouraged to refer children with motor delay (not sitting at 10 months, not standing at 12 months or walking at 24 months) for confirmation of a diagnosis of CP. Specialist clinics have been established within the Children's Health Services District where suitability for the study can be confirmed.

### Study entry

**Figure 2:**
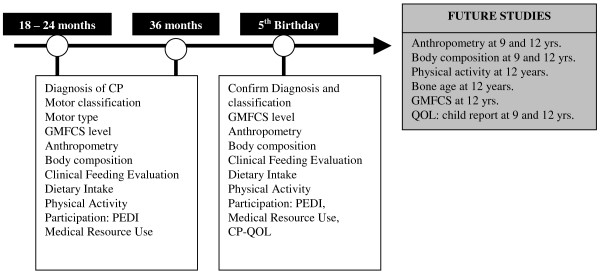
**Flow chart of study timeline & measures from baseline (left), to completion (right)**. GMFCS = the Gross Motor Function classification System; FMS = Functional Mobility Scale: TBW = Total Body Water; PEDI = The Paediatric Evaluation of Disability Inventory; CAPE = the Children's Assessment of Participation and Enjoyment; CP-QOL-child = the Cerebral Palsy Quality of Life measure (child version); Dx = Confirmed diagnosis.

Eligible children will enter the study from 18 months corrected age. They will be assessed for diagnostic criteria, co-morbidities and for differential neurological assessment by a Paediatric Rehabilitation Specialist and/or a Paediatric Neurologist. All measures will be performed on three occasions at 17 to 25 months (according to study entry); 36 ± 1 months and 60 ± 1 months corrected age (see Figure 2 flow chart for details). Children diagnosed after 25 months of age may enter the study at either 30 ± 1 months or 36 ± 1 months. To ensure collection of data at three time periods, these children will have their second assessment conducted at 48 ± 1 months. Written informed consent will be obtained from the parents or legal guardians prior to the commencement of data collection.

### Feasibility

Children who are detected after 18 months of age will be entered into the study at the time of diagnosis, will receive assessment at entry and be followed up until outcome at 5 years. According to the Queensland CP Register there are 80-120 new children with CP born in Queensland each year. We propose recruitment of at least 80 children each year (total 240 children from 360 potential children). High ascertainment is expected for children with moderate to marked motor delay (GMFCS III to IV) and this has been the case for children born preterm and children referred to the Queensland CP Health Service. Children born at term with mild motor delay (GMFCS I and II) and predominant lower limb involvement (diplegia) are typically identified through the Qld CP Health Service and CP Orthopaedic services at the Royal Children's and Mater Children's Hospitals.

Recruitment and data collection for this study is being conducted in conjunction with the Queensland CP Child Study of Motor Function and Brain Development (NHMRC 465128). This is a population based prospective cohort study (n = 240) which aims to determine the pathway(s) to motor outcome (gross and fine motor) from diagnosis at 18 months to outcome at 5 years in relation to the nature of the brain lesion (using structural MRI). Children enter the study at 18 months of age with assessments conducted every six months until 3 years of age then again at 4 years and final outcomes are assessed at 5 years.

### Measurements and procedures

#### Gross motor function

Gross Motor Function will be determined using the Gross Motor Function Measure (GMFM 66). The GMFM 66 has been shown to be valid and reliable and has been Rasch analysed to enable improved scaling [47]. Gross motor function assessment will be conducted by two experienced paediatric physiotherapists whom have criterion rating with the study developers (Boyd). All GMFM assessments will be video taped to enable scoring of the accelerometry data for validation of the Actigraph for the identification of time spent sedentary.

#### Motor type

Type of CP (eg, spastic, dystonic, or hypotonic) and motor distribution (unilateral, bilateral, number of limbs involved) will be determined by two independent physiotherapists at each assessment according to Sanger [46] and the internationally accepted classification system on the European CP Register [48]. The classification of motor type will be recorded for both physiotherapists, independently, at each assessment and common agreement will be assessed for rating motor type at a young age.

#### Functional severity

Functional severity will be determined using the internationally accepted Gross Motor Function Classification System (GMFCS) [34] by two independent physiotherapists trained in performing the gross motor function assessment. Children will be classified as being in one of five functional categories for the age bands under two years, two to four years and four to six years. The GMFCS has established validity and reliability for use in young children with CP [34,49]. Inter rater reliability for the current study will be determined.

#### Anthropometry

Weight will be measured to the nearest 100 grams using chair scales (Seca Ltd, Germany). Height or length will be measured to the last completed millimetre with a portable stadiometer/length measuring board (Shorr Productions, LLC, Maryland, USA). Knee height and upper-arm length will be measured with an anthropometer (Holtain Ltd, Dyfed, UK). Intraobserver reliability has technical errors of 0.23 cm for upper-arm length and 0.16 cm for knee height with coefficients of variation of 1.22% and 0.56% respectively [39]. Height estimates will be predicted from knee height and upper arm length using published validated equations [39]. Body mass index will be calculated as weight (kg) divided by height (m) squared. Head circumference and mid arm circumference will be measured to the last completed millimetre using a steel flexible measuring tape.

Duplicate measurements of tricep skin-fold thickness and subscapular skin-fold thickness will be measured using callipers (Holtain Ltd, Dyfed, UK) by trained investigators. By convention, all measurements will be conducted on the left side of the body. This protocol is modelled on the convention used for development of the National Centre for Health Statistics charts [50]. Data from skin-fold thicknesses have been found to be useful when assessing the nutritional status of children with CP [14]. Reliabilities have technical errors for intraobserver and interobserver measures of tricep skin-fold thickness of 0.60 mm and 0.55 mm with coefficients of variation of 5.93% and 6.98% respectively [7].

Anthropometric and body composition data will be converted to *Z*-scores using age and gender specific reference data for the general population [51,52]. Between-group comparisons will be conducted across GMFCS levels (I-V).

#### Body composition

Total body water (TBW) will be measured non-invasively, using the deuterium-dilution technique [53]. Children will be given a dose of deuterium in the form of water either orally or via feeding tube. In the absence of a feeding tube in children with feeding difficulties, children will be assessed to determine the most suitable technique to enable the consumption of the isotope with minimal risk of spillage. Any spillage that may occur will be collected in an absorbent cloth which will be weighed before and after dosing to accurately determine how much fluid has been lost [54]. A single baseline urine sample will be collected prior to administration of the dose to determine natural baseline enrichments of the isotopes and a second urine sample will be collected at approximately five hours after dosing. Measurement of the isotopic enrichment of a sample of body fluids at this time enables calculation of the body water pool using standard equations [55]. Collection of urine samples from children with poor or no bladder control will involve the inclusion of an absorbent liner in their nappy from which urine will be extracted for analysis [54]. Analyses of the urine samples will be performed using an isotope ratio mass spectrometer. Similar procedures have been used by our group and others in infants, children following severe traumatic brain injury and children with mild and severe CP [53,56-58]. The accuracy of TBW measured using the deuterium dilution technique is excellent at approximately 1% [59], and 1 - 2% for repeated measurements [60,61]. Fat free mass will be determined through division of TBW by age and gender specific hydration factors [42].

#### Bioelectrical impedance analysis

Impedance (Ohm) will be measured using a Body Stat 1500MDD (Isle of Mann, UK) at 800μA and a fixed frequency of 50 KHz. Children will be required to lie in a supine position with arms and legs slightly abducted from the trunk. The electrical current will be applied through two non-polarizing surface electrodes placed at the dorsal surfaces of the hand and foot over the distal aspect of the second and third metacarpals and metatarsals. The voltage drop will be measured by two further electrodes placed at the right pisiform prominence of the wrist and between the lateral and medial malleoli of the ankle. The proximal and distal electrodes will be a minimum of 5 cm apart. All measurements will be taken twice, with a third measurement taken if the difference is greater than 5 Ohm. The mean of the two closest values will be used for analysis. Total body water will be estimated from measurement of impedance and height or length using previously published equations [62-64]. The relationship between height^2^/impedance and TBW measured using deuterium dilution will be examined using regression analysis. An equation for the estimation of TBW from measures of height or length and impedance, specific for young children with CP, will be developed [53]. Reliability of measurements of impedance in this population will be determined.

#### Habitual time spent sedentary

The time that children spend sedentary in their own free-living environment will be measured using the ActiGraph GT3M accelerometer (Shalimar, FL). The GT3M is a small (3.8 × 3.7 × 1.8 cm), lightweight (27 g) triaxial accelerometer that detects accelerations of a magnitude and frequency that correspond with human movement, filtering out other forms of motion (e.g. vibration). Raw acceleration data is recorded in real time as counts per minute. Output from the device can be used to indicate when the wearer was active, as well as when they were sedentary.

Accelerometry is the most appropriate method for measurement of sedentary behaviour in this study. Self-report is inappropriate in this age-group as our pilot data demonstrate that parental report correlates poorly with criterion measures in the target population [65]. Pedometers measure only steps and are therefore inappropriate for use with children who do not walk, and doubly labelled water, while considered the gold standard for the measurement of physical activity, is prohibitively expensive and will not provide data on patterns of activity. Additionally, the ActiGraph has demonstrated cross-validity with criterion measures of activity in populations, age groups and activities relevant to the current study including: hip worn ActiGraphs for walking people with brain injury (r = 0.74) [66]; for measuring free play in young children (r = 0.72) [67]; and wrist worn ActiGraphs for measuring wheelchair activity in people with disabilities (r = 0.66) [68].

##### Time spent sedentary vs time spent active

While both time spent active and sedentary behaviour have established links with child health outcomes [36], our study will focus on measurement of sedentary behaviour. Time spent active will not be used as an outcome measure as young children with CP move in a variety of ways including walking, running, crawling, creeping, rolling, and bottom-shuffling. In combination with disordered movement kinematics and kinetics, these diverse modes of movement make the relationship between counts per minute and activity intensity for children with CP unpredictable. As a consequence, identification of time spent in moderate to vigorous physical activity (the intensity recommended for normal growth and development [36]) is impossible to derive from accelerometer output in this population. In contrast to accelerometer-based measurement of activity, we can be confident that, providing a child is wearing the monitor, if counts per minute are zero, the child is sedentary.

##### Identification of cut points for sedentary behaviour

There is a methodological challenge in choosing to measure sedentary behaviour: when counts per minute are greater than zero it does not necessarily follow that the child is active (e.g., very low but non-zero counts per minute will be registered with regular weight-shift that occurs with prolonged sitting). Therefore, in order to validly determine when a child has been sedentary, a criterion validity study will be conducted to determine cut-points for differentiating between sedentary behaviour and non-sedentary behaviour. The method used will be based on that described by Welk et al [69]

Participants in the criterion validation study for the Actigraph will be 100 children with CP participating in the Queensland CP Child Study of Motor Function and Brain Development, with a minimum of two children in each of the 15 possible combinations of age (17 to 25 months, 36 ± 1 months and 60 ± 1 months) and GMFCS level (I-V) in our sample. As part of their evaluation, children in the Queensland CP Child: Brain and Motor Development Study complete the Gross Motor Function Measure 66 [47], a standardised motor assessment battery which takes between 40-60 min to complete and requires the children to complete a range of motor tasks (e.g., sitting, standing, rolling, crawling etc). During these assessments, children will wear an Actigraph GT3M and will be video taped. The video of the assessment will subsequently be coded using BEST direct observation software to provide a real-time criterion measure of when the child was active and when they were sedentary. Active behaviour is defined as either positional change where the centre of gravity is moved (e.g., sit to stand, stand to sit, bending down) or translocation of any description (e.g., walking, crawling, rolling). Sedentary behaviour is defined as the child being stationary with or without limb or head movement. To derive unique sedentary cut-points (count per minute) which maximize sensitivity and specificity in each of the 15 cells, a receiver operator characteristic curve analysis will be conducted. For this analysis, counts per minute will serve as the independent variable, with a (1, 0) indicator variable corresponding to 1 = sedentary (as determined from direct observation) versus 0 = non-sedentary activity (again, as determined from direct observation) serving as the dependent variable.

##### Measurement of habitual time spent sedentary

To measure free-living sedentary behaviour at each of the three planned data collection points, ActiGraphs will be set for 15 second epochs and worn at the centre of the child's back [70], for a period of 3 days, the minimum required for a valid estimate of habitual activity in children [70]. Children will be required to wear the Actigraph during waking hours only and parents will be given instructions for wear and logging wear-time. After 3 days, the ActiGraph will be returned by courier for data extraction and analysis. Following return, output will be analysed for periods of non-wear and the data converted to mean counts per minute for the monitoring period. Analysis will be performed according to functional severity (GMFCS) with mean counts per minute used to stratify participants into high, medium and low levels of sedentary behaviour.

#### Dietary intake

Usual dietary intake will be determined using a three day weighed food record [71]. Parents will be instructed to weigh all food and fluids offered to the child before and after consumption. Parents will also be instructed to record information regarding the amount of food and fluids lost due to spillage as well as the time taken (in minutes) for the child to consume each meal, snack or drink. Food records will be reviewed by the Research Dietician with the caregiver present to clarify any ambiguous information. Food records will be analysed using the Foodworks™ dietary analysis software program (Xyris Software (Australia) Pty Ltd). Mean energy intake will be expressed as a percentage of age and gender specific recommendations [72].

#### Feeding ability

Oral motor and swallowing function will be assessed using a number of measures obtained from a parent completed feeding questionnaire, direct observation during a clinical feeding evaluation of a regular meal and from ratings derived from the video taped clinical feeding evaluation. Saliva control and drooling measures were derived from parent report and clinician's rating during the clinical feeding evaluation using a five point scale for severity and four point scale for frequency described by Thomas-Stonell and Greenberg [73]. A subset of clinical signs suggestive of aspiration will be noted from parent report in the feeding questionnaire and during the clinical feeding evaluation [74]. Objective measurement of oral motor function during feeding will be rated from the videotaped clinical feeding evaluation using the Schedule for Oral Motor Assessment (SOMA). The SOMA was normalised on 127 young infants aged 8-24 months with 10% of the population having CP. It has a positive predictive validity of 90% and sensitivity greater than 85% to detect clinically significant oral-motor dysfunction in infants and young children. This assessment has also been used to evaluate children of older ages. The SOMA has excellent levels of inter-rater reliability (kappa > 0.75) and intra-rater reliability (85%) [75-77]. Oral motor and swallowing function will also be formally rated using the Feeding and Swallowing Competency Subtest (Part 2) of the Dysphagia Disorders Survey (DDS) - Pediatric [17,78]. The DDS was developed as a screening tool to assess feeding and swallowing function in children and adults with developmental disability [3-78 years; mean 31.71 years] with 5% of the population aged 3-17 years (n = 31). It has more recently been used in a group of 166 children (2 years 1 month - 19 years 1 month; mean 9 years 4 months) with moderate to severe CP and intellectual disability [17,78]. Test validity and inter-item reliability were determined from a sample of 626 people with developmental disability. Inter-rater reliability was undertaken on a sample of 21 participants by 6 speech pathologists and achieved excellent reliability of 97% [17,78]. Inter-rater reliability of direct ratings for the SOMA and DDS will be compared for 10% of the participants in our study.

#### Participation

Participation will be determined using parent-report on the domains of self-care, mobility and social functioning using the scaled scores (rasch analysed) of the Pediatric Evaluation of Disability Inventory (PEDI) [79]. The PEDI is a generic standardised instrument of functional performance in children with disabilities that has been found to be both valid and reliable. It has been standardised on a sample of 412 able bodied American children between the ages of 0.5 and 7.5 years [79]. There are three independent domains of the PEDI (participation in self-care, mobility and social function) that are rated by parent report as capable (score = 1) or incapable to perform (score = 0). The PEDI has been found to be a valid and reliable assessment of functional performance in children with disabilities [79].

The mobility and self-care domain of the PEDI will be completed by the caregiver to assess the child's participation in activities of daily living. On the first occasion the PEDI will be administered as an interview (15-20 mins). On subsequent occasions it will be provided as a questionnaire mailed to the family for completion prior to the study visit, and will be checked by the researcher at the study visit. The PEDI raw aggregate scores can be converted into normative standard scores and scaled scores using conversion tables provided in the manual [79]. Scaled scores provide an indication of the child's performance along a continuum of item difficulty or complexity in a particular domain. The range of possible scores (0-100) represents increasing levels of function. In the present study, all raw scores will be converted to scaled scores (Rasch analysed) to compare the entire group (age range 18 months to five years) of all children across the self-care domain for capability, without the difficulties of 'ceiling and floor' effects due to age limitation in the normative standard scores.

#### Quality of life

Parent perception of QOL will be assessed using the condition specific tool CP QOL-child (CP QOL-Child) from 4 years of age [80]. The CP QOL-Child assesses aspects of life that parents and children have identified as important including physical wellbeing, social wellbeing, emotional wellbeing, school, access to services, and acceptance by others. The psychometric properties of the CP QOL - Child are excellent with Cronbach's Alpha range from 0.74-0.92 for parent-proxy report [80]. Test re-test is adequate, where ICC 0.76-0.89 and it is moderately correlated with generic QOL and health (r = 0.30-0.51) [80]

#### Resource use and the direct costs of treatment

In order to determine the relationship between motor prognosis and resource use, medical and allied health resource use and the direct costs of treatment will be monitored and compared to outcomes with adjustment for confounders such as disease severity using cost and consequences analysis [81].

### Sample size calculations

240 children will be studied with three measurements planned for each participant between 18 months and 5 years of age. For hypothesis 1, a sample size of 45 per group (GMFCS I-V, totalling 225 patients) will have 80% power and 5% significance of detecting a between group difference in height of 6 cm (assuming a standard deviation of change of 10 cm) between 18 months and 5 years of age, between functional groups and non-CP infants [52]. To allow for attrition we will enrol 240 infants in total.

### Statistical considerations

Primary analysis will use the intention to treat principle, using the Last Observation Carried Forward principle for participants who withdraw before the end of the study period. Differences between participants who complete and withdraw will be assessed using t-tests for continuous variables, after transformations of non-normally distributed variables, and Fisher's Exact Test for categorical variables. Baseline characteristics of the GMFCS groups will be compared similarly. Details for the statistical models that will be used to analyse data to address each hypothesis are as described below.

#### H1

Outcome is attainment of GMFCS, a 5-level categorical variable at 5 yrs. We will consider the explanatory variables of growth and nutritional status in separate models. Individual Z-scores for height or predicted height from knee height or upper arm length will be determined at 18, 36 and 5 yrs and modelled using mixed-effects models. These models are used as they incorporate both fixed and random variables in the analysis. We will model using a random-intercept and slope for each participant. We will test potential covariates (eg sex) and include them as fixed effects if appropriate.

#### H2

Outcome is attainment of GMFCS at 5 years. Explanatory variables are fat free mass and fat mass at 18, 36 and 5 years. We will investigate the association between explanatory and outcome variables using separate mixed-effects models.

#### H3a

Outcome variables are growth status and body composition. We will investigate the association with explanatory variables of dietary intake and habitual physical activity at 3 years and 5 years. We will use mixed-effects models with random intercept and slope for each participation, with GMFCS as a fixed effect and with appropriate interaction terms.

#### H3b

Outcome variables are growth status, nutritional status and body composition at 5 years of age. Explanatory variables are dietary intake and time spent sedentary at 18 months of age. We will investigate the ability of the explanatory variables to predict the outcome variables using mixed-effects models.

#### H4

Outcomes are health care utilization and direct medical costs, participation (PEDI) at 3 and 5 years and QOL at 5 yrs. Explanatory variables are growth, body composition and time spent sedentary at 18 months and 36 months. We will investigate the association between explanatory and outcome variables using mixed-effects models with a random intercept and slope for each participant, and functional severity at 18 months included as a fixed effect.

## Competing interests

The authors declare that they have no competing interests.

## Authors' contributions

PSWD, RNB, KLB, SMT, KAW and RDS contributed to the study design, study protocol and grant writing. KLB modified the grant for publication with input from all coauthors. All authors read and approved the final manuscript.

## Pre-publication history

The pre-publication history for this paper can be accessed here:

http://www.biomedcentral.com/1471-2458/10/179/prepub

## References

[B1] Stanley F Blair E Alberman E Cerebral Palsies: Epidemiology and Causal Pathways 2000 151 London: Mac Keith Press

[B2] Rosenbaum P Paneth N Leviton A Goldstein M Bax M A report: The definition and classification of cerebral palsy April 2006 Dev Med Child Neurol 2007 49 S8 14 17370477

[B3] Access Economics Pty Limited The Economic Impact of Cerebral Palsy in Australia in 2007 2008

[B4] Stevenson RD Hayes RP Cater LV Blackman JA Clinical Correlates of Linear Growth in Children with Cerebral Palsy Dev Med Child Neurol 1994 36 135 142 8132124 10.1111/j.1469-8749.1994.tb11822.x

[B5] Krick J Murphy Miller P Zeger S Wright E Pattern of Growth in Children with Cerebral Palsy J Am Diet Assoc 1996 96 680 685 10.1016/S0002-8223(96)00188-5 8675911

[B6] Rogozinski BM Davids JR Davis RB Christopher LM Anderson JP Jameson GG Blackhurst DW Prevalence of Obesity in Ambulatory Children with Cerebral Palsy J Bone Joint Surg Am 2007 89 2421 2426 10.2106/JBJS.F.01080 17974884

[B7] Stevenson RD Conaway M Chumlea WC Rosenbaum P Fung E Henderson CJ Worley G Liptak GS O'Donnell M Samson Fang L Growth and Health in Children with Moderate to Severe Cerebral Palsy Pediatr 2006 118 1010 1018 10.1542/peds.2006-0298 16950992

[B8] Day SM Strauss DJ Vachon PJ Rosenbloom L Shavelle RM Wu YW Growth Patterns in a Population of Children and Adolescents with Cerebral Palsy Dev Med Child Neurol 2007 49 167 171 10.1111/j.1469-8749.2007.00167.x 17355471

[B9] Stallings VA Charney EB Davies JC Cronk CE Nutrition-Related Growth Failure of Children with Quadriplegic Cerebral Palsy Dev Med Child Neurol 1993 35 126 138 8444326 10.1111/j.1469-8749.1993.tb11614.x

[B10] Rempel GR Colwel SO Nelson RP Growth in Children with Cerebral Palsy fed via Gastrostomy Pediatr 1988 82 857 862 3186375

[B11] Henderson CJ Lovell DJ Specker BL Campaigne BN Physical Activity in Children with Juvenile Rheumatoid Arthritis: Quantification and Evaluation Arthritis Care Res 1995 8 114 119 10.1002/art.1790080210 7794985

[B12] Stevenson RD Roberts CD Vogtle L The Effects of Non-Nutritional Factors on Growth in Cerebral Palsy Dev Med Child Neurol 1995 37 124 130 7851668 10.1111/j.1469-8749.1995.tb11981.x

[B13] Troughton KE Hill AE Relation Between Objectively Measured Feeding Competence and Nutrition in Children with Cerebral Palsy Dev Med Child Neurol 2001 43 187 190 11263689

[B14] Fung EB Samson Fang L Stallings VA Conaway M Liptak GS Henderson RC Worley G O'Donnell M Calvert R Rosenbaum P Feeding Dysfunction is Associated with Poor Growth and Health Status in Children with Cerebral Palsy J Am Diet Assoc 2002 102 368 373 10.1016/S0002-8223(02)90084-2 11902369

[B15] Sanders KD Cox K Cannon R Blanchard D Pitcher J Papathakis P Varella L Maughan R Growth Response to Enteral Feeding by Children with Cerebral Palsy J Parenter Enter Nutr 1990 14 23 26 10.1177/014860719001400123 2109109

[B16] Sullivan PB Juszczac E Bachlet A Lambert B Vernon-Roberts A Grant H Eltumi M Mclean L Alder N Thomas A Gastrostomy tube feeding in cerebral palsy: a prospective, longitudinal study Dev Med Child Neurol 2005 47 77 85 10.1017/S0012162205000162 15707230

[B17] Calis EA Veugelers R Sheppard JJ Tibboel D Evenhuis HM Penning C Dysphagia in children with severe generalized cerebral palsy and intellectual disability Dev Med Child Neurol 2008 50 625 630 10.1111/j.1469-8749.2008.03047.x 18754902

[B18] Tahmassebi JF Curzon ME The cause of drooling in children with cerebral palsy -- hypersalivation or swallowing defect? Int J Paediatr Dent 2003 13 106 111 10.1046/j.1365-263X.2003.00439.x 12605628

[B19] Reilly S Skuse D Characteristics and Management of Feeding Problems of Young Children with Cerebral Palsy Dev Med Child Neurol 1992 34 379 388 1592191 10.1111/j.1469-8749.1992.tb11449.x

[B20] Gisel EG Alphonce E Classification of eating impairments based on eating efficiency in children with cerebral palsy Dysphagia 1995 10 268 274 10.1007/BF00431421 7493509

[B21] Gisel EG Applegate-Ferrante T Benson J Bosma JF Oral-motor skills following sensorimotor therapy in two groups of moderately dysphagic children with cerebral palsy: aspiration vs nonaspiration Dysphagia 1996 11 59 71 10.1007/BF00385801 8556880

[B22] Arvedson J Rogers B Buck G Smart P Msall M Silent aspiration prominent in children with dysphagia Int J Pediatr Otorhinolaryngol 1994 28 173 181 10.1016/0165-5876(94)90009-4 8157416

[B23] Rogers B Arvedson J Buck G Smart P Msall M Characteristics of dysphagia in children with cerebral palsy Dysphagia 1994 9 69 73 10.1007/BF00262762 8131428

[B24] Rogers BT Arvedson J Msall M Demerath RR Hypoxemia during oral feeding of children with severe cerebral palsy Dev Med Child Neurol 1993 35 3 10 8449378 10.1111/j.1469-8749.1993.tb11545.x

[B25] Burg J van der Jongerius P van Limbeek J van Hulst K Rotteveel J Drooling in children with cerebral palsy: a qualitative method to evaluate parental perceptions of its impact on daily life, social interaction, and self-esteem Int J Rehabil Res 2006 29 179 182 10.1097/01.mrr.0000194395.64396.f1 16609333

[B26] Burg JJ Van der Jongerius PH Van Hulst K Van Limbeek J Rotteveel JJ Drooling in children with cerebral palsy: effect of salivary flow reduction on daily life and care Dev Med Child Neurol 2006 48 103 107 10.1017/S0012162206000235 16417664

[B27] Burg JJ van der Jongerius PH van Limbeek J van Hulst K Rotteveel JJ Social interaction and self-esteem of children with cerebral palsy after treatment for severe drooling Eur J Pediatr 2006 165 37 41 10.1007/s00431-005-1759-z 16172877

[B28] Pena AH Cahill AM Gonzalez L Baskin KM Kim H Towbin RB Botulinum toxin A injection of salivary glands in children with drooling and chronic aspiration J Vasc Interv Radiol 2009 20 368 373 10.1016/j.jvir.2008.11.011 19157908

[B29] Lodha R Puranik M Natchu UC Kabra SK Recurrent pneumonia in children: clinical profile and underlying causes Acta Paediatr 2002 91 1170 1173 10.1080/080352502320777388 12463313

[B30] Seddon PC Khan Y Respiratory problems in children with neurological impairment Arch Dis Child 2003 88 75 78 10.1136/adc.88.1.75 12495971 PMC1719284

[B31] Reilly JJ Thoraya HM Braekken A Jolly J Day RE Growth Retardation and Undernutrition in Children with Spastic Cerebral Palsy J Hum Nutr Diet 1996 9 429 435 10.1046/j.1365-277X.1996.00468.x

[B32] Samson-Fang L Fung E Stallings VA Conaway M Worley G Rosenbaum P Calvert R O'Donnell M Henderson RC Chumlea WC Relationship of Nutritional Status to Health and Societal Participation in Children with Cerebral Palsy J Pediatr 2002 141 637 643 10.1067/mpd.2002.129888 12410191

[B33] Liptak G O'Donnell M Conaway M Chumlea W Health status of children with moderate to severe cerebral palsy Dev Med Child Neurol 2001 43 364 371 10.1017/S001216220100069X 11409824

[B34] Palisano R Rosenbaum P Walter S Russell D Wood E Galuppi B Development and Reliability of a System to Classify Gross Motor Function in Children with Cerebral Palsy Dev Med Child Neurol 1997 39 214 223 9183258 10.1111/j.1469-8749.1997.tb07414.x

[B35] Stephenson J Bauman A Armstrong T Smith B Bellew B The costs of illness attributable to physical inactivity in Australia 2000 Canberra, Australia

[B36] Department of Health and Aging Australia's Physical Activity Recommendations for 5-12 year olds. Canberra 2004

[B37] Weil E Wachterman M McCarthy E Davis R O'Day B Lezzoni L Wee C Obesity among adults with disabling conditions J Am Med Assoc 2002 288 1265 1268 10.1001/jama.288.10.1265 12215134

[B38] Australian Bureau of Statistics Sport and Recreation: A Statistical Overview 2006 Canberra, Australia

[B39] Stevenson RD Use of Segmental Measures to Estimate Stature in Children with Cerebral Palsy Arch Pediatr Adolesc Med 1995 149 658 662 7767422 10.1001/archpedi.1995.02170190068012

[B40] Chumlea WC Prediction of Stature from Knee Height for Black and White Adults and Children with Application to Mobility-Impaired or Handicapped Persons J Am Diet Assoc 1994 94 1385 1390 10.1016/0002-8223(94)92540-2 7963188

[B41] Bell KL Davies PSW Prediction of Height from Knee Height in Children with Cerebral Palsy and Non-Disabled Children Ann Hum Biol 2006 33 493 500 10.1080/03014460600814028 17060071

[B42] Fomon SJ Haschke F Ziegler EE Nelson SE Body Composition of Reference Children from Birth to Age 10 Years Am J Clin Nutr 1982 35 1169 1175 7081099 10.1093/ajcn/35.5.1169

[B43] Lohman T Applicability of Body Composition Techniques and Constants for Children and Youths Exerc Sport Sci Rev 1986 14 325 355 10.1249/00003677-198600140-00014 3525188

[B44] Butte NF King JC Energy requirements during pregnancy and lactation Public Health Nutrition 2005 8 1010 1027 16277817 10.1079/phn2005793

[B45] Elia M Ritz P Stubbs RJ Total energy expenditure in the elderly Eur J Clin Nutr 2000 54 Suppl 3 S92 103 11041080 10.1038/sj.ejcn.1601030

[B46] Sanger T Delgado M Gaebler Spira D Hallett M Mink J Classification and definition of disorders causing hypertonia in childhood Pediatr 2003 111 e89 e97 10.1542/peds.111.1.e89 12509602

[B47] Russell DJ Rosenbaum PL Cadman DT Gowland C Hardy S Jarvis S The gross motor function measure: a means to evaluate the effects of physical therapy Dev Med Child Neurol 1989 31 341 352 2753238 10.1111/j.1469-8749.1989.tb04003.x

[B48] Surveillance of Cerebral Palsy in Europe Surveillance of Cerebral Palsy in Europe: A Collaboration of Cerebral Palsy Surveys and Registers Dev Med Child Neurol 2000 42 816 824 10.1017/S0012162200001511 11132255

[B49] Palisano RJ Hanna SE Rosenbaum PL Russell DJ Walter SD Wood EP Raina PS Galuppi BE Validation of a Model of Gross Motor Function for Children with Cerebral Palsy Phys Ther 2000 80 974 985 11002433

[B50] Owen G Measurement, recording and assessment of skinfold thickness in childhood and adolescence: report of a small meeting Am J Clin Nutr 1982 35 629 638

[B51] Addo O Himes J Reference curves for triceps and subscapular skinfold thicknesses in US children and adolescents Am J Clin Nutr 2010 91 635 642 10.3945/ajcn.2009.28385 20053877

[B52] Kuczmarski RJ Ogden CL Grummer-Strawn LM CDC Growth Charts: United States Advance Data from Vital and Health Statistics, no. 314, Hyattsville, MD 2000 11183293

[B53] Bell KL Davies PSW The Use of Bioelectrical Impedance Analysis in Children with Cerebral Palsy and Non-Disabled Children Int J Body Comp Res 2004 2 15 22

[B54] Atkin LM Davies PSW Diet Composition and Body Composition in Preschool Children Am J Clin Nutr 2000 72 15 21 10871555 10.1093/ajcn/72.1.15

[B55] Halliday D Miller AG Precise Measurement of Total Body Water Using Trace Quantities of Deuterium Oxide Biomed Mass Spectrom 1977 4 82 87 10.1002/bms.1200040205 884210

[B56] Sullivan PB Alder N Bachlet A Grant H Juszczac E Henry J Vernon-Roberts A Warner J Wells J Gastrostomy feeding in cerebral palsy: too much of a good thing Dev Med Child Neurol 2006 48 10.1017/S0012162206001927 17044953

[B57] Bandini LG Schoeller DA Fukagawa NK Wykes LJ Dietz WH Body Composition and Energy Expenditure in Adolescents with Cerebral Palsy or Myelodysplasia Pediatr Res 1991 29 70 77 10.1203/00006450-199101000-00014 2000262

[B58] Stallings VA Zemel BS Davies JC Cronk CE Charney EB Energy Expenditure of Children and Adolescents with Severe Disabilities: A Cerebral Palsy Model Am J Clin Nutr 1996 64 627 634 8839510 10.1093/ajcn/64.4.627

[B59] Schoeller DA van Santen E Peterson DW Dietz WH Jaspan J Klein PD Total Body Water Measurement in Humans with ^18^O and ^2^H Labeled Water Am J Clin Nutr 1980 33 2686 2693 6776801 10.1093/ajcn/33.12.2686

[B60] Racette S Schoeller D Luke A Relative dilution spaces of 2H and 18O labeled water in humans Am J Physiol 1994 267 E585 590 7943308 10.1152/ajpendo.1994.267.4.E585

[B61] Wells JCK Fuller NJ Dewit O Fewtrell MS Elia M Cole TJ Four-Component Model of Body Composition in Children: Density and Hydration of Fat-Free Mass and Comparison with Simpler Models Am J Clin Nutr 1999 69 904 912 10232629 10.1093/ajcn/69.5.904

[B62] Pencharz PB Azcue M Use of Bioelectrical Impedance Analysis Measurements in the Clinical Management of Malnutrition Am J Clin Nutr 1996 64 485S 488S 8780368 10.1093/ajcn/64.3.485S

[B63] Kushner RF Schoeller DA Fjeld CR Is the Impedance Index (Ht^2^/R) Significant in Predicting Total Body Water? Am J Clin Nutr 1992 56 835 839 1415001 10.1093/ajcn/56.5.835

[B64] Fjeld CR Freundt-Thurne J Schoeller DA Total body water measurement by ^18^O dulution and bioelectrical impedance in well and malnourished children Pediatr Res 1990 27 98 102 10.1203/00006450-199001000-00024 2104972

[B65] Bell KL Energy Expenditure, Body Composition and Physical Activity Levels of Children with Cerebral Palsy PhD Thesis 2005 University of Queensland, Discipline of Paediatrics and Child Health, School of Medicine

[B66] Tweedy S Trost SG Validity of accelerometry for measurement of activity in people with brain injury Med Sci Sports Exerc 2005 37 1474 1480 10.1249/01.mss.0000177584.43330.ae 16177597

[B67] Kelly L Reilly J Fairweather S Barrie S Grant S Payton J Comparison of two accelerometers for assessment of physical activity in preschool children Ped Exerc Sci 2004 16 324 333

[B68] Washburn R Copay A Assessing physical activity during wheelchair pushing: validity of a portable accelerometer Adapted Physical Activity Quarterly 1999 16 290 299

[B69] Welk G Eisenmann J Schaben J Trost S Dale D Calibration of the biotrainer pro activity monitor in children Ped Exerc Sci 2007 19 145 158 10.1123/pes.19.2.145 17603138

[B70] Trost S McIver K Pate R Conducting accelerometer-based activity assessments in field-based research Med Sci Sports Exerc 2005 31 s531 543 10.1249/01.mss.0000185657.86065.98 16294116

[B71] Sullivan PB Juszczac E Lambert B Rose M Ford Adams M Johnson A Impact of feeding problems on nutritional intake and growth: Oxford feeding study II Dev Med Child Neurol 2002 44 461 467 10.1017/S0012162201002365 12162383

[B72] National Health and Medical Research Council Nutrient Reference Values for Australia and New Zealand Including Recommended Dietary Intakes. Canberra 2005

[B73] Thomas-Stonell N Greenberg J Three treatment approaches and clinical factors in the reduction of drooling Dysphagia 1988 3 73 78 10.1007/BF02412423 3271655

[B74] Weir K McMahon S Barry L Masters IB Chang AB Clinical signs and symptoms of oropharyngeal aspiration and dysphagia in children Eur Respir J 2009 33 604 611 10.1183/09031936.00090308 19010985

[B75] Reilly S Skuse D Mathisen B Wolke D The objective rating of oral-motor functions during feeding Dysphagia 1995 10 177 191 10.1007/BF00260975 7614860

[B76] Reilly S Skuse D Wolke D Schedule for oral motor assessment 2000 London: Whurr

[B77] Skuse K Stevenson J Reilly S Mathisen B Schedule for oral-motor assessment (SOMA): Methods of validation Dysphagia 1995 10 192 202 10.1007/BF00260976 7614861

[B78] Sheppard J Dysphagia Disorders Survey and Dysphagia Management Staging Scale, Users Manual and Test Forms revised. Netherlands edition 2002 Lake Hopatcong, NJ: Nutritional Management Associates

[B79] Hayley SM Coster S Ludlow L Haltwinger J Andrellos P The Pediatric Evaluation of Disability Inventory (PEDI): Development, standardisation and administration manual 1992 Boston: New England Medical Centre and PEDI Research Group

[B80] Davis E Shelly A Waters E Boyd R Cook K Casey E Reddihough D The impact of caring for a child with cerebral palsy: Quality of life for mothers and fathers Child: Care, Health and Development 2008 10.1111/j.1365-2214.2009.00989.x 19702639

[B81] Houltram J Noble I Boyd RN Corry I Flett P Graham HK Botulinum toxin type A in the management of equinus in children with cerebral palsy: an evidence-based economic evaluation Eur J Neurol 2001 8 194 202 10.1046/j.1468-1331.2001.00052.x 11851748

